# Knowledge mapping of immune thrombocytopenia: a bibliometric study

**DOI:** 10.3389/fimmu.2023.1160048

**Published:** 2023-05-03

**Authors:** Feifeng Wu, Cuifang Li, Jueyi Mao, Junquan Zhu, Yang Wang, Chuan Wen

**Affiliations:** Department of Pediatrics, The Second Xiangya Hospital, Central South University, Changsha, China

**Keywords:** immune thrombocytopenia, bibliometric analysis, citespace, VOSviewer, research hotspot and trends

## Abstract

**Background:**

Immune thrombocytopenia (ITP) is an autoimmune disease characterized by isolated thrombocytopenia. Recently, the pathophysiology and novel drugs of ITP have been the focus of researchers with plenty of publications emerging. Bibliometrics is the process of extracting measurable data through statistical analysis of published research studies to provide an insight into the trends and hotspots.

**Objective:**

This study aimed to provide an insight into developing trends and hotspots in the field of ITP by bibliometric analysis.

**Methods:**

By using three bibliometric mapping tools (bibliometrix R package, VOSviewer, CiteSpace), we summarized the overview information of retrieved publications, as well as the analysis of keyword co-occurrence and reference co-citation.

**Results:**

A total of 3299 publications with 78066 citations on ITP research were included in the analysis. The keyword co-occurrence network identified 4 clusters relating to the diagnosis, pathophysiology, and treatment of ITP respectively. Then the reference co-citation analysis produced 12 clusters with a well-structured and highly credible clustering model, and they can be divided into 5 trends: second-line treatment, chronic ITP, novel therapy and pathogenesis, COVID-19 vaccine. Treg cells, spleen tyrosine kinase, and mesenchymal stem cells were the latest hotspots with strong burstness.

**Conclusion:**

This bibliometric analysis provided a comprehensive insight into research hotspots and trends on ITP, which would enrich the review of the ITP research.

## 1 Introduction

Immune thrombocytopenia (ITP) is an autoimmune disease characterized by isolated thrombocytopenia. Patients may be asymptomatic at presentation, or they may present with mild mucocutaneous or life-threatening bleeding (1). The pathophysiology of ITP is complex and remains incompletely understood. The classical theory stated that platelet destruction and impaired megakaryocyte maturation were caused by autoantibodies (2). In addition, T-cell-mediated cytotoxicity toward platelets and megakaryocytes has been proven to be one of the important mechanisms (3, 4). In recent years, the pathogenesis of ITP has been further explored and found to be extensive, encompassing cellular immunity, humoral immunity, and bone marrow microenvironment (2). The emergence of new drugs, such as thrombopoietin receptor agonists (TPO-RAs), made the treatment of ITP no longer limited to immune suppression and splenectomy (5). The research aimed to explore a new pathology and treatment of ITP has gained increased attention with many publications.

The rapid progress and development of medical science in recent years have led to an exponential growth of academic papers every year, resulting in so-called information overload or filtering failure (6). Scholars often found it difficult to grasp the trends and hotspots of research in a huge literature base. Bibliometrics is the process of extracting measurable data through statistical analysis of published research studies and shows how the knowledge within a publication is used, providing a broad synthesis of a research field and demonstrating how it has changed over time, as well as identifying influential studies or scholars (6). It can not only enable researchers to gain a clearer overview of the research on a given topic but also identifies research abundancies, gaps, and trends of potential moderators (7). Thus, bibliometric analysis was wildly used in various fields in the last decade. Recently, Yang Ou et al. performed a bibliometric analysis of primary ITP from 2011 to 2021, which provided novel insights for researchers on future research trends and scientific decision-making (8). However, in the first decade of the 21st century, there have been some breakthroughs in the exploration of the pathogenesis and treatment of ITP, such as CD8+ T cells and TPO-RAs, and these documents were significant for understanding the lineage and direction of the field (3, 9). Encouraged by previous studies, we performed a bibliometric analysis of the literature concerning ITP for the last 20+ years (1999–2022), hoping to provide a comprehensive analysis of the research trends and hotspots.

## 2 Materials and methods

The data used in this study were retrieved from the Web of Science Core Collection database (WoSCC) on October 16, 2022. The search keyword query was: TI= “Idiopathic Thrombocytopenic Purpura*” OR “Immune Thrombocytopenic Purpura*” OR “Immune Thrombocytopenia*” OR “Werlhof* Disease” OR “Autoimmune Thrombocytopenia*” OR “Autoimmune Thrombocytopenic Purpura*” OR ITP. The database source was limited to the Web of Science Citation Index Expanded (SCI-Expanded) with a publication date from January 1, 1999, to October 16, 2022. Original articles and reviews were included as document types since they can represent the current status of research in a field (10, 11). The language was limited to English. Then, the “full record and cited references” of the retrieved documents were extracted in text files. Two of the authors reviewed all titles and abstracts independently to determine their eligibility. In case of disagreement, it was solved through discussion or further recourse to a third person. Before further bibliometric analysis, Python programming language was used for data cleaning, mainly to remove duplicates and replace synonyms. In addition, some results were presented in two parts to make them more rational: basic science papers (mainly including *in vivo* and *in vitro* experiments exploring the pathology and mechanisms of ITP) and clinical papers (mainly including guidelines, clinical trials, case reports, etc.).

Many bibliometric mapping software tools have been created and used by scholars. In this study, we chose the widely used tools for the analysis (12). For descriptive analysis, we used the bibliometrix R package (13) to analyze the amounts of publications, citations, and the trends of growth, as well as top authors, sources, cited sources, and their impact. We used several indicators, including the number of publications, the average number of citations, the h-index, and the g-index, to assess the impact of authors. The h-index is the highest number of papers that received h or more citations among a set of papers, while the g-index is the highest number g of papers that together received g^2^ or more citations (14, 15). Both two indicators can reflect the publication (quantity) and citation (quality). For network mapping, VosViewer (16) (version 1.6.18; http://www.VOSviewer.com/) was used to analyze the co-occurrence of keywords and co-authorship between countries/regions. The networks of co-cited references and the burstness of keywords were analyzed by CiteSpace (17) (version 6.1.3; http://cluster.ischool.drexel.edu/~cchen/citespace/).

## 3 Results

### 3.1 The overview of publications

**Figure 1:**
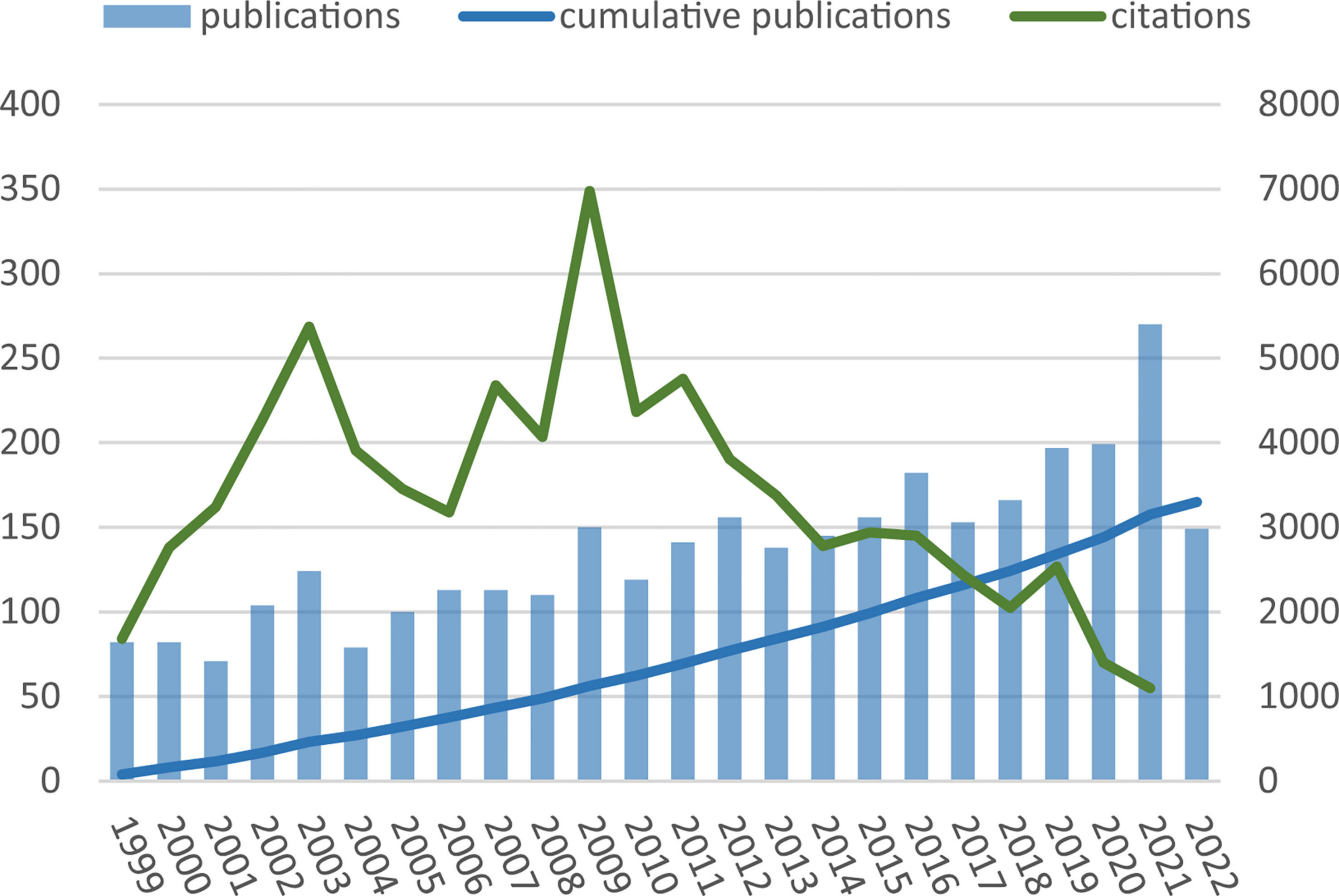
Annual publications and citations over time.

The search in the WoSCC database resulted in a total of 3,402 publications, including original articles and reviews, without duplications. After two authors assessed eligibility, 104 documents unrelated to ITP were excluded, and finally, 3299 articles were included in the analysis, comprising 808 basic research papers and 2491 clinical papers. The main information about all documents and the two subgroups was shown in **Table 1**. During the recent decades, both publications and citations had a steady increase, with annual publication numbers peaking in 2021 (**Figure 1**). The average growth rate of the literature was 3.27% over the last 20 years. The annual growth rate of basic science papers was higher than that of clinical papers at 8% and 2.31%, respectively. By 2021, the cumulative number of citations was 78066, and the average number of citations per document was 23.59, with similar citation frequencies between the two subgroups (21.1 and 24.4, respectively).

**Table 1:** Main information about data.

	All docs	Basic science docs	Clinical docs
Timespan	1999:2022	1999:2022	1999:2022
Sources	679	215	583
Documents	3299	808	2491
Annual growth rate %	3.27	8	2.31
Document average age	10.6	9.24	11
Average citations per doc	23.59	21.1	24.4
References	38840	16035	26610
Authors	12475	3489	10047
Authors of single-authored docs	114	7	111
Single-authored docs	183	9	174
Co-authors doc	6.69	7.82	6.33
International co-authorship %	13.64	13.99	13.53

### 3.2 Authors, institutions, regions, and sources of publications

**Figure 2:**
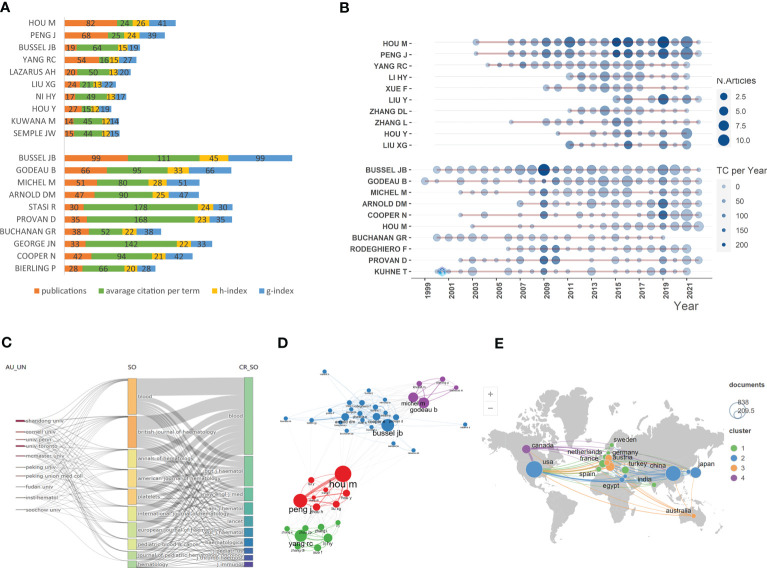
Descriptive statistical analyses of publications on ITP. **(A)** Publications and impact of the most-contributing authors in basic science papers (top) and clinical papers (bottom). **(B)** Top authors’ production over time in basic science papers (top) and clinical papers (bottom). **(C)** A Sankey diagram between institutions, sources and cited sources. **(D)** Co-authorship network of the authors in the field of ITP. **(E)** Global geographical distribution and network visualization map of country/region collaborations.

The total number of authors retrieved in the documents was 12,475, with an average of 6 authors per article. A total of 75.8% of the authors wrote only one article (**Figure S1**). The ten most productive and influential authors in the two groups were presented in **Figure 2A**. In general, the most-productive author in the basic science group was Hou M, who published 82 papers with 24 citations per doc and also had the highest g-index and h-index. On the other hand, Bussel JB published the highest number of clinical articles. The top authors’ production over time was presented in **Figure 2B**. The co-authorship network showed that Peng J and Hou M collaborated closely, and the two scholars focused on the pathogenesis of ITP (18–20). In addition, two collaborative groups centered around Bussel JB and Godeau B, respectively, significantly contributed to clinical trials and management guidelines (**Figure 2D**) (21, 22).

We also analyzed the main institutions and sources implicated in the field. As shown in **Figure 2C**, four of ten highly productive institutions were in China, with Shandong University topping the ranking, indicating that institutions in China played a crucial role in ITP research. A total of 3299 papers related to ITP were published in a wide range of 679 journals. *Blood* and *British Journal of Haematology* contributed a large portion of the ten journals with the greatest number of ITP-related articles, both of which belong to the Journal Citation Reports (JCR) “hematology” category. Meanwhile, references cited in the documents were mainly from the two journals. The top ten productive and locally cited sources were shown in **Table S1**, **S2** respectively. According to the global geographical distribution and network visualization map, most papers were published by authors from North America and East Asia (**Figure 2E**). Further, the United States of America (USA) contributed the most published papers, followed by China and Japan. The international cooperation visualization map showed that USA collaborated most closely with China and Japan, while collaborations among other countries were scattered.

### 3.3 Keyword co-occurrence analysis

**Figure 3:**
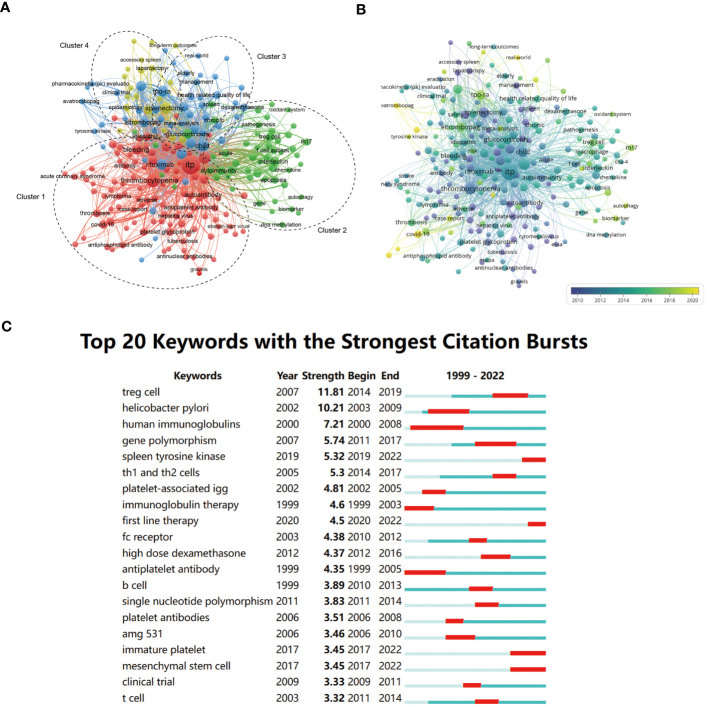
network visualization **(A)**, overlay visualization **(B)**, and top citation burst **(C)** of keyword co-occurrence analysis. **(A)** In the network visualization, items (nodes) are represented by different keywords. The size of the nodes is determined by the number of occurrences and the color represented the cluster to which the item belongs. In general, the closer two nodes are located to each other, the stronger their relatedness. All nodes are divided into four clusters (dotted circles). **(B)** An overlay visualization based on the average publication year. The size and position of the nodes are the same as **(A)**. The color of the nodes represents the average publication year, and the corresponding relationship is shown in the legend. **(C)** The top 20 keywords with the strongest citation bursts. The red bold line represents the burst years.

Author keywords, an essential part of an article given by the authors, can represent the research topic of a publication. To explore the research trends and hotspots, 279 out of 2610 keywords with a frequency of 5 and above were extracted for the co-occurrence analysis in VOSviewer. The 60 most frequently occurring keywords were shown in **Table 2**. In the network visualization, keywords could be classified into 4 clusters (**Figure 3A**). Cluster 1 (red) was the largest cluster mainly related to the diagnosis and manifestations of ITP, where the nodes were close to the center of the network (such as “bleeding,” “thrombocytopenia,” and “autoantibody”) and closely distributed. Cluster 2 (green) focused on basic research on the pathological mechanisms of ITP, including keywords such as “T cell subset,” “Treg cell,” “Th17 cell,” “interleukin,” “DNA methylation,” “apoptosis,” etc. Cluster 3 (blue) primarily referred to the management of ITP as evidenced by keywords “glucocorticoids,” “rituximab,” “spleen tyrosine kinase,” “splenectomy,” “eltrombopag,” etc. The last cluster (yellow) was small and only included a few keywords, such as “splenectomy.”

**Table 2:** High occurrences keywords in ITP research.

Keyword	Freq.	Keyword	Freq.	Keyword	Freq.
ITP	2016	cytokine	60	chronic	34
thrombocytopenia	351	Treg cell	59	flow cytometric analysis	33
platelet	243	autoimmunity	57	pediatric	33
splenectomy	229	autoimmune disease	55	CD markers	31
child	191	gene polymorphism	50	antiplatelet antibody	30
b-cell depletion	168	megakaryocyte	48	prednisolone	30
TPO-RAs	151	COVID-19	45	Th17 cell	30
bleeding	140	human immunoglobulins	45	anti-d	29
immunoglobulin therapy	132	outcome	45	diagnosis	29
eltrombopag	125	pregnancy	41	apoptosis	28
romiplostim	116	thrombosis	41	immune	28
chronic ITP	114	HRQOL	40	Fc receptor	27
glucocorticoids	108	platelet glycoprotein	40	safety	27
platelet examination	87	T cell	38	B cell	26
thrombopoeitin	79	dexamethasone	37	T cell subset	26
interleukin	77	vaccine	37	infection	25
helicobacter pylori	75	idiopathic	36	autoimmune	24
purpura	66	predictor	36	epidemiology	24
treatment	66	ICH	35	interferon	24
autoantibody	64	SLE	35	adult	723

ITP, immune thrombocytopenia; TPO-RAs: thrombopoietin receptor agonists; HRQOL, health related quality of life; ICH, intracerebral hemorrhage; SLE, systemic lupus erythematosus.

We plotted the overlay visualization for the co-occurring author keyword network to analyze the trends of the study. The color of an item was determined by the average publication year, where colors ranged from blue (earlier) to green and yellow (latest). It showed that keywords such as “COVID-19,” “avatrombopag,” and “tyrosine kinase” were newly emerging research topics. “Th17 cell,” “TPO-RAs,” and “autophagy” were hotspots in latest five years. In addition, the analysis of burstness (**Figure 3C**) revealed that “Treg cell” was the keyword with the strongest burst in 2014–2019. When focusing on the last five years, more attention was paid to “spleen tyrosine kinase,” “first-line therapy,” “immature platelet,” and “mesenchymal stem cells.”

### 3.4 Reference co-citation analysis

**Figure 4:**
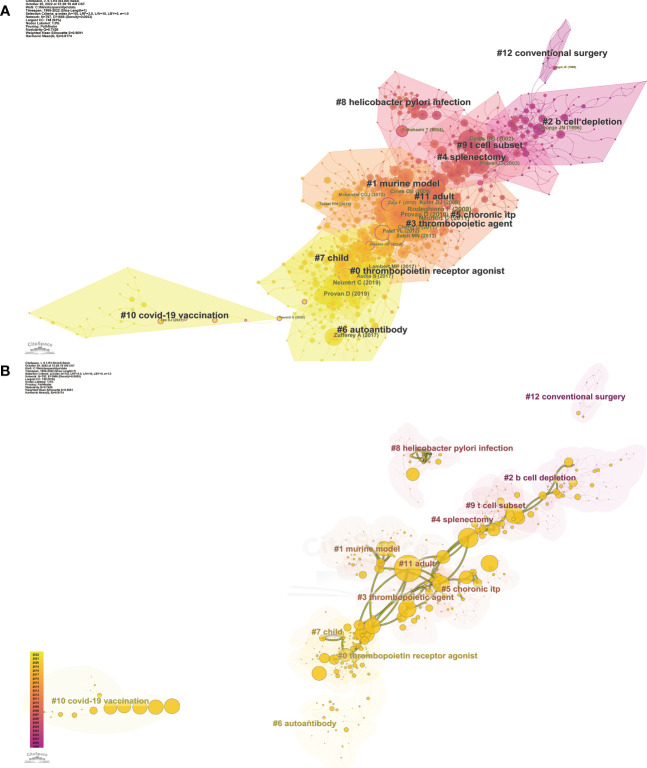
Co-citation references network **(A)** and correspondent betweenness centrality analysis **(B)** by CiteSpace. **(A)** The network with cluster visualization indicated different research hotspots. The nodes presented different references, the size and color of a node are determined by the co-citation frequency and publication year respectively. Annual citations of each reference are rendered as citation tree rings. The most recent citations correspond to the innermost rings. The network is decomposed into clusters of references based on the strengths of co-citation links. Clusters are numbered in descending order of their size. The largest one is numbered as #0, followed by #1, and so on. **(B)** The Visualization map with overlays of main paths (red) and core references (yellow) is organized by betweenness centrality, revealing the key hubs to connect different clusters. The centrality scores are normalized to the unit interval of [0,1], and the higher the score, the larger the node. The color of a link represented the earliest time slice in which the connection was first made.

In general, bibliometric outcomes included citation, co-citation, and co-occurrence. Co-citation is defined as when two published articles are cited together by another publication. We further extracted the co-citation network using CiteSpace (**Figure 4**). The network presented a high Q value (Q = 0.7426) and a t silhouette score (S = 0.9091), indicating a well-structured and highly credible clustering model (23). Finally, 12 clusters were identified and could be further classified into five research trends.

The first trend started with cluster #12 conventional surgery and evolved to cluster #2 B cell depletion. The trend mainly included publications relating to the second-line treatment of ITP. It shared hotspots with and then gradually evolved to clusters #4, #5, and #9. Conventional splenectomy was once considered the first choice for drug-resistant chronic ITP (cITP). Many patients received surgery and achieved remission (24). In the 1990s, many scholars compared laparoscopic surgery with conventional surgery, suggesting the superiority of laparoscopic splenectomy in terms of rapid recovery and short hospital stay (25, 26). Otherwise, children with ITP were less likely to undergo splenectomy. However, due to the potential adverse effects including the operative complications and the risk of fatal bacterial infection (27), the place of splenectomy as the second-line treatment has been gradually challenged by other therapy approaches, such as B cell depletion (28).

The second trend was a topic concerning chronic ITP, including 4 clusters: #5 chronic ITP, #9 T cell subset. #4 splenectomy, and #8 *Helicobacter pylori* infection. Approximately 25% to 30% of adult patients developed cITP that was refractory to corticosteroids and splenectomy and other available treatments (28). The cause of cITP is not clear and may be related to some risk factors. Several groups have recently reported that eradication of *Helicobacter pylori* led to platelet recovery in patients with cITP, but little was known about the pathogenesis of cITP. Takahashi T et al. have further elucidated the role of molecular mimicry and cross-reactivity between PAIgG and *H. pylori* CagA protein (29). Even though the exact mechanism remained unclear, multiple guidelines have recommended eradication therapy of *H. pylori* in patients with cITP (30, 31). The immune imbalance of T cell subsets was the focus of pathogenic mechanism research and thought to be strongly associated with cITP. This was consistent with the hotspots of cluster 2 in the keyword network analysis. Among these studies, regulatory T cells (Treg cells), helper T cells (Th cells), and other T cell subsets were well studied (32).

Next, the two main trends were closely related to each other, where both the number and size of nodes significantly increased, suggesting that the research on ITP was in full swing with plenty of publications published and cited. The clusters here could be divided into clinical and basic research. The former included cluster #11 adult, #3 thrombopoietic agent, #7 child, and #0 TPO-RA, mainly including clinical trials and management guidelines for different patient groups. The latter included cluster #1 murine model and #6 autoantibody, suggesting that the pathogenesis of ITP has always been a focused area.

The last and latest trend discussed the topic of vaccination, which was introduced by a single cluster #10 COVID-19 vaccination. The COVID-19 pandemic has led to urgent development and widespread use of SARS-CoV-2 vaccines. Vaccines are crucial for protecting vulnerable groups, such as the old, the young, and the patients suffering from other diseases. Meanwhile, due to the potentially higher risk of adverse reactions, the indication of vaccination in this population has become the focus of public attention. Thrombocytopenia caused by vaccination was reported as early as the 20th century (33, 34). Recently, it has been reported that ITP may be identified *de novo* after SARS-CoV-2 vaccination, and the condition may worsen in part of preexisting ITP, especially in post-splenectomy patients and those with more refractory disease (35). Practical guidance has been formulated for the assessment and management of patients with ITP during the COVID-19 pandemic (36).

### 3.5 Developing trend analysis

**Figure 5:**
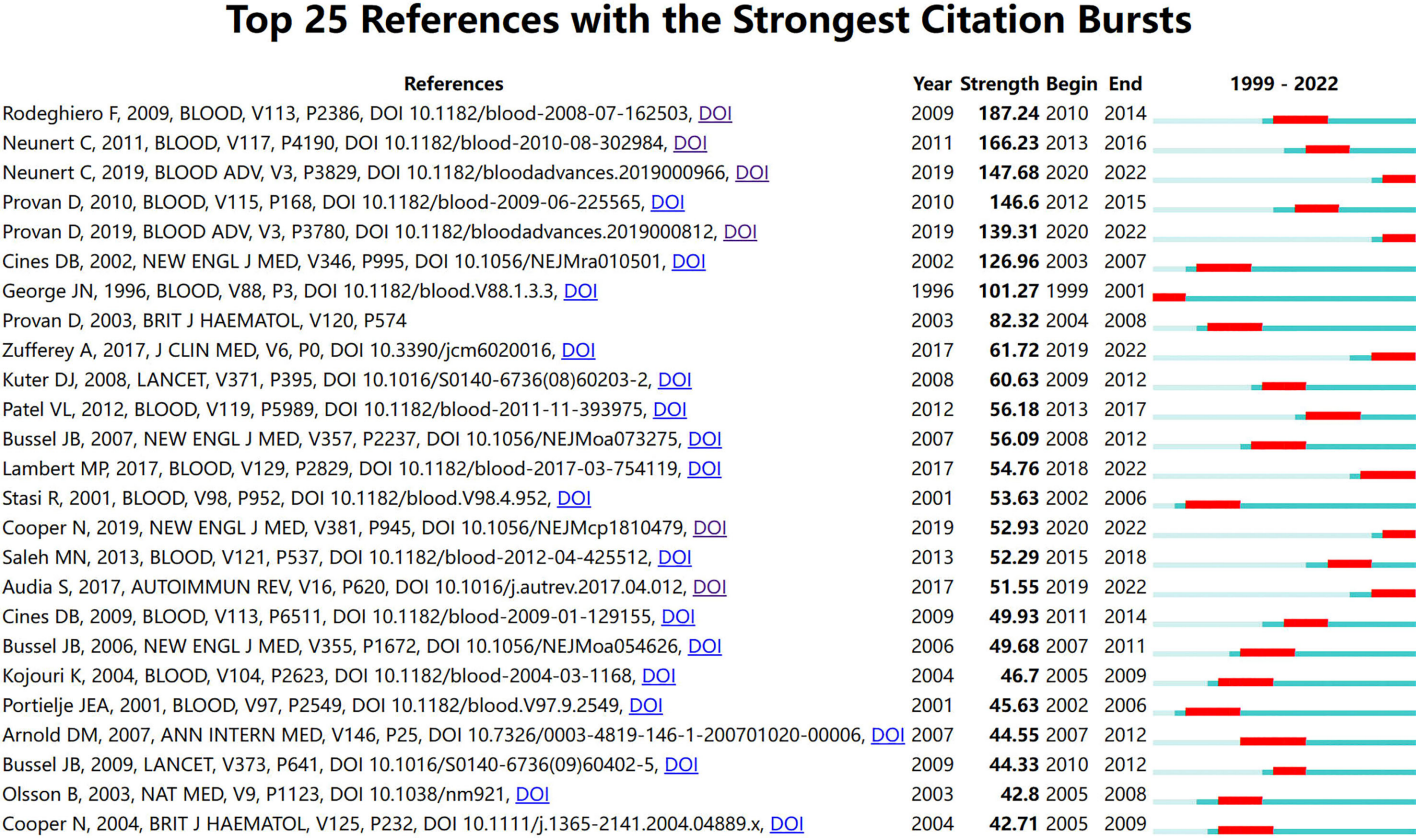
The top 25 references with the strongest citation bursts. The red bold line represents the burst years.

Furthermore, we introduced temporal metrics, citation burstness, to measure the rate of change in a specific duration, thus, identifying emergent terms. The top 25 references with the strongest citation bursts were presented in **Figure 5**, in which the former items were all focused on guidelines for ITP from different organizations (31, 37, 38). In addition, reviews related to pathological mechanisms have recently also received more attention (39, 40). Among the basic research papers, the work that received a high citation burst for the first time illustrated the role of CD8+ cytotoxic T cells in platelet destruction, with important implications for elucidating the pathogenesis of ITP (3). The overlays of main paths and core references were presented in **Figure 4B** to analyze the developing trend. Nodes with high betweenness centrality generally connect different clusters and are considered key hubs, exerting a great influence on a network (7). Among these nodes, some references were highly connected to other nodes and positioned between different clusters, which could be seen as landmark works in the context of the research area of ITP. It was evidenced that the trend of ITP was based on the exploration of new therapies; meanwhile, research on related pathogenesis was always on the way. Throughout the development of ITP research, clinical and basic topics have been closely integrated and progressed together. There has been a remarkable temporal evolution in the treatment of ITP, from splenectomy and B-cell depletion therapy at the beginning of the 21st century to thrombopoietic agents and TPO-RAs in the last decade.

## 4 Discussion

Our research enriched the review of the research field of ITP. Based on the bibliometric analysis, it could be concluded that the study of novel therapy and pathogenesis of ITP were the main research hotspots. Beginning with the study showing that the infusion of plasma from patients with ITP to healthy volunteers would lead to thrombocytopenia (41), the study on the pathophysiology of ITP has never ceased. In the above-mentioned analysis, Treg cells and Th cells (mainly Th17 and Th1) were topics with high frequency and strong bursts, indicating that their role in ITP has been intensely investigated. The significance of Th17 cells was confirmed in a previous bibliometric analysis study about the ITP field in the last decade (8). Notably, our results showed that Treg cells also attracted much attention in T cell subsets. Since the first study suggested that lack of T cell suppression was responsible for platelet destruction, an impressive output of papers showed that T cell homeostasis played a predominant role (42). Treg cells, which were responsible for immune tolerance, have been studied the most during a long period, and these cells were deficient in ITP and failed to suppress the excessive auto-reactive T cells (43, 44). Furthermore, the imbalance of Th cells has been evidenced by an increasing polarization of Th1 and Th17 cells, as well as their cytokines (45). Increased Th17 cells and related cytokines have been evidenced in ITP patients and mice and correlated with the course of the disease and response to treatment (46–48).

A better understanding of the pathophysiology has promoted the development of new drugs. Over the decades, immunosuppression has been the essential method in traditional therapy of ITP, such as glucocorticoids and immunoglobulin. There are often some patients who are resistant to these treatments or suffer from the side effects of long-term medication. The decision regarding the second-line treatment for ITP has been debated for a long time. Recently, many new alternative drugs have emerged, among which TPO-RAs has been the most popular. As early as the 20th century, researchers began to explore novel therapy to replace or reduce the use of immunosuppressive drugs, such as the first-generation TPO mimetic drugs and recombinant human TPO. Although it did increase platelet counts, its clinical efficacy was limited as it may be neutralized by antibodies in patients (49). In 2008, the second-generation TPO mimetic drugs, eltrombopag and romiplostim, were licensed in the USA for the treatment of ITP, and since then, their use has progressively increased around the world (5, 50). TPO-RAs were well-tolerated and effective drugs for ITP with acceptable toxicity, which have been soon recommended by specialists as second-line therapy for ITP and even as first-line treatment in some patients (31, 51, 52). Besides, other new agents that function by preventing FcyR-mediated platelet destruction were also attractive and have been proven for clinical or advanced phase, including spleen tyrosine kinase (Syk) inhibitors, Bruton tyrosine kinase inhibitors (BTKIs), and neonatal Fc receptor inhibitors (2). Among them, Syk, one of the hot spots of research in recent years, was involved in FcγR-mediated platelet destruction and has been considered a therapeutic target for ITP (53). Its inhibitor Fostamatinib was approved by the FDA in 2018 as a new second-line agent for the treatment of ITP (54). Finally, mesenchymal stem cells (MSCs) have been highly studied in autoimmune diseases due to their immunomodulatory ability. Similarly, it has recently attracted attention in the ITP field (**Figure 3C**). Impaired bone marrow MSCs play an important role in the pathogenesis of ITP (55, 56). Meanwhile, the infusion of MSCs to patients with refractory ITP has also been supported by clinical evidence of encouraging efficacy (57, 58). Thus, MSCs have great potential in ITP, which is worthy of further exploration.

This study had some limitations. Compared to narrative reviews, bibliometric analysis can handle thousands of publications to comprehensively reveal the developing trends and topics of research. However, since the analysis method is based on the publications and their references, it is inevitably affected by various biases, such as publication bias and citation bias (59). The results may sometimes be biased in the keywords-based analysis because some keywords of papers are not as accurate. For example, regarding Th17 cells, a cell of high interest to scholars, the frequency of this keyword was only 30, which was much lower than the number of articles retrieved from the database. Second, the data analyzed in this study were only retrieved from the WoSCC, leading to the relative incompleteness of the retrieved publication. Nevertheless, the WoSCC has been broadly accepted among researchers and was considered a common tool for both retrieving and bibliometric analysis due to its high quality and integrity of data (60, 61). Besides, the article type and language of publications were limited, which might have led to the same concern.

Despite the limitations, this study also had strengths. First, this study provided a bibliometric analysis of the literature in the ITP field over the last 20 years, which would contribute to the understanding of research hotspots and developing trends in this field. Compared with previous research, we merged the synonyms in the keywords before the analysis, which could minimize the information omission caused by the scattered distribution of keywords due to synonyms. In addition, the analysis in this study was conjunctively achieved by three widely-used tools (CiteSpace, VOSviewer, and R package) for better visualization. Furthermore, the bibliometric outcomes of this study included citation, co-citation, and co-occurrence, and different types of visualization mapping were drew to comprehensively elucidate the research field. We studied multiple bibliometric indicators (for example, citations, g index, and h index were employed to evaluate the impact), temporal metrics, such as citation burstness, and structural metrics, such as betweenness centrality.

This bibliometric analysis provided a comprehensive insight into research hotspots and trends on ITP. Both publications and citations have witnessed a steady increase over the decades. USA, China, and Japan were the countries that studied ITP most and worked closely with each other. In the further analysis of 3299 publications, the research on pathophysiology and treatment of ITP was identified as the research hotspot, such as TPO-RAs, T cell subsets, etc. In addition, COVID-2019, Syk, and MSCs were emerging hot issues. Therefore, this study can help researchers better explore the development and trends of this field.

## Data availability statement

The original contributions presented in the study are included in the article/**Supplementary Material**. Further inquiries can be directed to the corresponding author.

## Author contributions

FW: Formal analysis and writing-original draft. CL: Data curation and validation. JM: Software and methodology. JZ and YW: Conceptualization. CW: Reviewing and editing, supervision. All authors contributed to the article and approved the submitted version.
