# Supplementary figures and images for: Knowledge mapping of immune thrombocytopenia: a bibliometric study
Source: Front Immunol. 2023 May 3;14:1160048. doi: 10.3389/fimmu.2023.1160048 (PMC10189105; doi:10.3389/fimmu.2023.1160048)

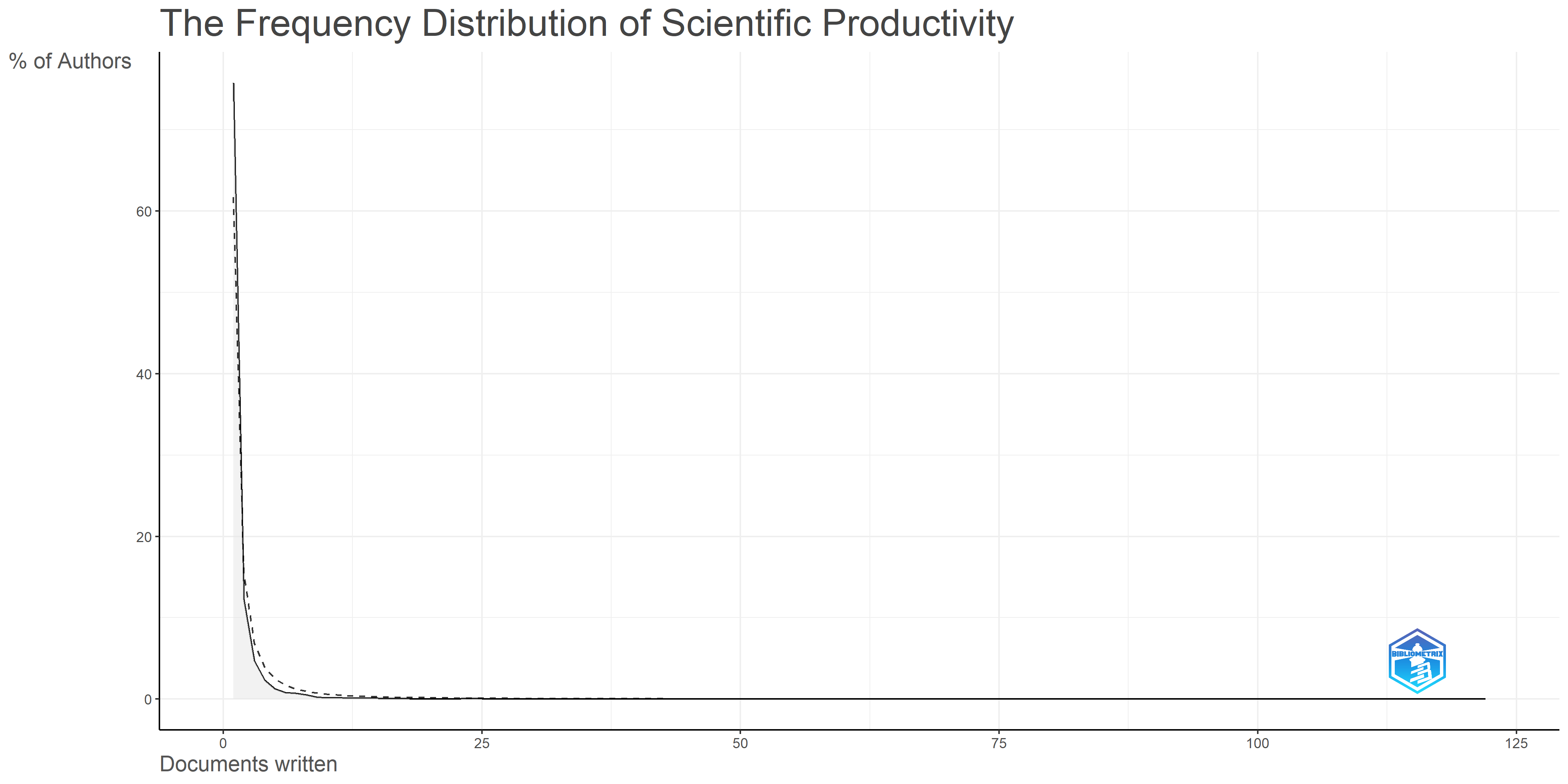

Supplement: Supplementary file 1 [file Image_1.png]
