# Supplementary material for: Knowledge mapping of immune thrombocytopenia: a bibliometric study
Source: Front Immunol. 2023 May 3;14:1160048. doi: 10.3389/fimmu.2023.1160048 (PMC10189105; doi:10.3389/fimmu.2023.1160048)
Supplement: Supplementary file 2 [file Table_1.docx]

**Table S1 Top 10 relevant sources**

| **Sources** | **Articles** |
| --- | --- |
| British Journal OF Haematology | 164 |
| Blood | 158 |
| Platelets | 112 |
| American Journal of Hematology | 110 |
| Annals of hematology | 97 |
| International Journal of Hematology | 95 |
| Pediatric Blood & Cancer | 91 |
| European Journal of Haematology | 88 |
| Journal of Pediatric Hematology Oncology | 87 |
| Hematology | 58 |

**Table S2 Top 10 local cited sources**

| **Sources** | **Articles** |
| --- | --- |
| Blood | 18155 |
| British Journal OF Haematology | 7006 |
| New England Journal of Medicine | 3333 |
| American Journal of Hematology | 2947 |
| Lancet | 2406 |
| European Journal of Haematology | 2095 |
| haematologica | 1911 |
| Journal of Pediatrics | 1392 |
| Journal of Thrombosis and Haemostasis | 1391 |
| Journal of Immunology | 1340 |
